# Group performance differences in reading, math, and science by fourth graders with and without communication disorders

**DOI:** 10.1590/2317-1782/e20250322en

**Published:** 2026-07-31

**Authors:** Courtney Karasinski

**Affiliations:** 1 Grand Valley State University – GVSU - Grand Rapids (MI), United States of America.

**Keywords:** Language Disorder, Academic Achievement, Mathematics, Science, Reading

## Abstract

**Purpose:**

Theoretical perspectives of learning academic subjects and curricular standards highlight the critical role of language ability. Thus, it is important to assess the academic achievement of children with language deficits to ensure the development of interventions to facilitate success in the classroom. This investigation aimed to compare the performance of fourth graders with communication disorders (ComDis) to those with typical language development (GenEd) on measures of reading, mathematics, and science achievement. Interaction effects of gender and income with language status were examined.

**Methods:**

Data were from an extant dataset, the Early Childhood Longitudinal Study Kindergarten Class of 2010-11. ANOVA compared performance on measures of reading, mathematics, and science in ComDis and GenEd fourth graders and the relation of gender and income.

**Results:**

For all three subjects, GenEd students scored higher than ComDis students, and upper-income students earned significantly higher scores than middle-income students, who earned significantly higher scores than low-income homes. Mathematics was a relative strength for children across communication status, income, and gender. A female advantage was found in reading, but not in mathematics or science.

**Conclusion:**

Communication deficits and low-income status adversely impact mastery of academic content. It is critical that speech-language pathologists collaborate with content-area teachers to ensure academic success for these children.

## INTRODUCTION

Language ability plays a critical role in the mastery of academic subjects. Language is the medium through which instruction is delivered, creating challenges for individuals who have difficulty with language comprehension^(1,2)^. Theoretical perspectives of learning highlight this crucial role of language ability in mastery of reading^(3)^, math^(2,4)^ and science^(5)^. Current educational standards in the United States, where this investigation was conducted, including the Common Core State Standards (CCSS) for English Language Arts & Literacy^(6)^, the CCSS Mathematics Standards for Mathematical Practice^(7)^, and the Next Generation Science Standards^(8)^, emphasize the importance of language ability in the core subject areas. Similarly, in the United Kingdom, the national curriculum^(9)^ and in Brazil, the Base Nacional Comum Curricular (BNCC) highlight the role of language across subjects^(10)^. Yet, despite the fundamental role language plays in learning each school subject, relatively few studies have empirically investigated the performance of children with language disorders across the academic curriculum. The current investigation compares fourth-grade students with and without communication disorders in their performance on measures of grade-level reading, math, and science. It includes the roles of gender and income level, given the literature on gender and socioeconomic differences in both communication ability and academic performance.

Prior research has indicated that children with language impairment perform more poorly than typically developing children in language arts, math, and science. For example, Durkin et al.^(11)^ found that language scores significantly predicted performance in English, math, and science at age 11. They revealed that English was the most difficult of the three subjects for students with language impairment. Science was a relative strength for the children with language impairment, and math ability fell between English and science proficiency. Five-year-old children who had been identified as late talkers as toddlers earned significantly lower scores on measures of reading and math compared with typically developing peers^(12)^. Phonological awareness and oral language comprehension at age five are significant predictors of language and mathematics in 4^th^, 7^th^, and 10^th^ grades^(13)^.

Children with language impairment perform more poorly than typically developing peers on aspects of mathematics including number transcoding, counting, arithmetic, and story problems^(1)^. Mathematical difficulties in children with language impairment may be due to difficulty with manipulating mathematical symbols, using working memory to recognize patterns, and using complex linguistic syntax^(14)^.

To date, few studies have investigated the performance of individuals with communication disorders on measures of achievement in science. One of the few studies comparing students with and without language disorders revealed that sixth graders with language/literacy disorders earned lower scores on measures of science achievement and science vocabulary breadth and depth^(15)^. More studies examining the performance in science of children with communication disorders are clearly needed. Scores on language tasks, including understanding narratives, non-fiction passages, and complex syntax, have been found to predict scores on measures of science achievement^(16)^.

### Theoretical perspectives

In response to the continued academic difficulties of many students, despite educational reforms, Shanahan and Shanahan^(17)^ proposed the following model of the increasing specialization of literacy development. The foundation is *basic literacy*, typically mastered during the primary grades, which includes recognizing high-frequency words, decoding words with regular spelling patterns, and understanding print conventions. By the end of middle school, most students master the next level, *intermediate literacy*, which includes general comprehension, understanding common word meanings, decoding lower-frequency words, comprehension monitoring, and fluency. The top level is comprised of *disciplinary literacy*, which refers to the specialized literacy skills needed for specific subjects, such as math or science. These skills are more challenging to learn because there are fewer parallels to oral language; texts in math and science may be abstract or ambiguous and may not relate to the students’ own experiences; and these skills usually are not taught explicitly. Students begin to master disciplinary literacy during middle school and high school, but many never master the skills that facilitate success in math and science. This theory highlights the role of language ability as an underlying factor in mastering the literacy of a specific discipline, which is necessary for successful learning and achievement.

### The simple view of reading

The relation between oral and written language ability is widely accepted. The simple view of reading posits that reading consists of two dissociable processes: decoding and linguistic comprehension^(3)^. *Decoding* refers to word recognition and is phonologically based. *Linguistic comprehension* involves using semantic word knowledge and integrating this knowledge with the syntactic knowledge of the sentence form to make discourse-level interpretations. The simple view of reading has been used to inform the differential diagnosis of dyslexia, poor reading comprehension, and specific language impairment, and is the basis of the national curriculum in the United Kingdom^(9)^. Bishop and Snowling^(18)^ described oral language deficits and reading deficits as having similar behavioral characteristics, but different causal mechanisms. Individuals with classic dyslexia, specific language impairment, and poor reading comprehension exhibit different profiles of language deficits. Children with specific language impairment may show deficits in a single aspect of or, more likely, a combination of syntax, morphology, phonology, semantics and pragmatics. In contrast, individuals with dyslexia, a disorder in decoding written words, show deficits only on phonology, and individuals with poor reading comprehension do not evidence phonological deficits, but do display deficits in the other areas of language

#### Mathematics

The role of language ability in learning mathematics is evident in theories of how math is learned. For example, Sfard^(4)^ described the *communicational approach to cognition* as applied to mathematics education. She noted that “communication should be viewed not as a mere aid to thinking, but as almost tantamount to the thinking itself” (p.13). This model emphasizes 1) individuals’ participation in their own learning, 2) conceptualizing thinking as communicating, 3) learning as initiation to a discourse, 3) mediating tools, including language, and 4) meta-discursive rules. This model is based on a socio-cultural perspective that integrates communicative interactions into specific contexts. It highlights the central role that mathematics discourse plays in mathematical learning. Similarly, Peng and colleagues’^(2)^ *developmental function hypothesis of language for mathematics* emphasizes the role of language in learning math, noting that language is used as a medium for communicating, representing, and retrieving math knowledge and to activate working memory and reasoning processes when performing mathematical tasks and learning math.

#### Theoretical perspectives on learning science

Research in science education emphasizes the role of language in the study of science^(5,19,20)^. Conferences have been held for the specific purpose of exploring the role of language in science education^(20)^, as science educators recognize that, as Hand and colleagues^(19:848)^ state, “There is no science without language”.

Yore and Treagust^(20:292)^ highlight the importance of *scientific discourse*, which includes “oral, written, symbolic, and physical modes of scientific language”. Bae and colleagues^(21:864)^ note that, “providing students with ongoing opportunities to make sense of science ideas through talk is at the heart of science learning”.

*Science literacy* includes both *fundamental* science literacy, which refers to reading and writing science content, and *derived* science literacy, which refers to knowledge, learning, and education^(5)^. Norris and Phillips^(5)^ argue that reading and writing are constitutive parts of science, rather than merely tools for doing science. They emphasize constructing meaning from text, rather than merely decoding text, as critical to science literacy. They also note the importance of oral language in conjunction with written language.

Based on these conversations in the science education research community, investigations have empirically evaluated the outcomes of integrating language and science teaching, with positive results. For example, results of a three-year longitudinal study of the Science Writing Heuristic approach to science teaching, which embeds language opportunities into science classrooms, revealed increases in students’ scores in measures of both science and language^(19)^.

### Curricular standards highlighting the role of language in core subjects

The American Speech-Hearing Association^(22)^ states that speech-language pathologists’ (SLPs’) roles in schools should include addressing the linguistic and metalinguistic foundations of the curriculum for students with disabilities and students at risk for poor academic performance. Thus, it is critical that SLPs understand the intersection of language ability and curricular standards. Language is integrated into the teaching of the content areas; both the CCSS^(6,7)^ and the NGSS^(8)^ place language at the forefront of education in the United States, as does the national curriculum in the United Kingdom^(9)^ and the BNCC^(10)^ in Brazil.

The CCSS *English Language Arts & Literacy (ELA)* standards explicitly integrate written and oral language comprehension and production^(6)^. Although the standards for *speaking and listening*, *reading*, *writing*, and *language* are separated, the introduction to the standards explains that this was done to facilitate clear understanding, but that these processes are inextricably linked^(6:4)^. The ELA standards were designed to apply to a myriad of subjects. The CCSS state that students who master the ELA standards can apply these skills to the learning of new content areas^(6:7)^; this description highlights the role of language in learning across disciplines.

The CCSS *Mathematics Standards for Mathematical Practice*^(7)^ clearly depend on linguistic processes. The first three standards are (1) make sense of problems and persevere in solving them, (2) reason abstractly and quantitatively, and (3) construct viable arguments and critique the reasoning of others. The role of language in each of these standards is clear from their titles. The sixth standard, *attend to precision*, also depends on language ability, as the description of this standard explicitly states the importance of communicating clearly and using accurate definitions. Standards seven and eight, *look for and make use of structure* and *look for and express regularity in repeated reasoning*, also relate to language ability, given the role of language in verbal reasoning. The *Standards for Mathematical Content* include a balance of procedure and comprehension. A number of these standards use metalinguistic verbs, including *understand* and *describe*, highlighting role of language comprehension and production in the mastery of mathematics content.

The NGSS consist of three dimensions: science and engineering practices, crosscutting concepts, and disciplinary core ideas^(8)^. Karasinski^(16)^ described the role of language in the NGSS, noting that each of the eight components of science and engineering practices and three of the seven components of crosscutting concepts are contingent on linguistic processes. The third dimension, disciplinary core ideas, corresponds with Shanahan and Shanahan’s^(17)^ description of disciplinary literacy, emphasizing the use of specific language abilities to master the subject content.

In Brazil, the BNCC^(9)^ highlight the role of language across subjects. The fourth of 10 core competencies in the BNCC is, “To use different languages ​​– verbal (oral or visual-motor, such as Libras, and written), bodily, visual, sound, and digital – as well as knowledge of artistic, mathematical, and scientific languages, to express oneself and share information, experiences, ideas, and feelings in different contexts and produce meanings that lead to mutual understanding.”^(23)^ The eighth competency is, “To argue based on facts, data, and reliable information, to formulate, negotiate, and defend ideas, points of view, and common decisions that respect and promote human rights, socio-environmental awareness, and responsible consumption at the local, regional, and global levels, with an ethical stance regarding self-care, care for others, and care for the planet.”^(23)^ These fourth and eighth competencies clearly emphasize the crucial role of language ability in the demonstration of learning and knowing. The standards in the subject areas include more specific ways that language is essential for the mastery of these subjects. For example, in science, students are expected to communicate about scientific concepts using different languages and forms of expression (e.g., EF03CI05). In mathematics, students produce written texts to summarize conclusions, discuss the purpose of research, and synthesize results (e.g., EF05MA24, EF05MA25).

### Gender and socioeconomic differences in academic achievement

In the United States, socioeconomic achievement gaps in reading, science, and math are evident at kindergarten entry, and remain stable throughout the school years^(24)^. Data from the United States Department of Health and Human Services National Institutes of Health Magnetic Resonance Imaging Study of Normal Brain Development revealed that poverty was associated with structural differences in the brain in areas associated with school readiness, including total gray matter, the frontal and temporal lobes, and the hippocampus. The children in poverty earned lower scores on behavioral tests of intelligence and language and math achievement^(25)^.

Investigations into the contribution of gender to language ability and academic achievement have been conducted in several countries using large longitudinal datasets. Studies reveal that females often earn higher scores on measures of language ability or academic achievement than their male peers^(26)^.

### Aims of the current investigation

The current investigation used a publicly available extant dataset, Early Childhood Longitudinal Study, Kindergarten Class of 2010-11 (ECLS-K:2011), to compare the performance of fourth graders with and without communication disorders on math, reading, and science measures. The ECLS-K:2011, sponsored by the National Center for Educational Statistics (NCES), includes a nationally representative sample of children enrolled in kindergarten in the United States during the 2010-11 academic year. The primary aim of the current study was to identify differences in academic achievement in three core subjects: reading, math, and science, by children with and without communication disorders. Identifying gender differences in academic achievement in children with and without communication disorders was a secondary aim for two reasons. First, gender differences in performance in the academic subjects have been evidenced in typically developing children in numerous countries^(26)^, with a small but significant female advantage, particularly in language coursework. Second, the prevalence of language impairment is higher in boys (8%), than in girls (6%)^(27)^, suggesting a female advantage in language ability. Income level was included in the analyses, given the achievement gap between learners of upper- and lower-income backgrounds that has long been observed in academics. SLPs have an important role in working toward the prevention of adverse academic outcomes^(22)^. Thus, it is important to evaluate the role of income in performance on measures of achievement in reading, math, and science to facilitate early identification of children who may need support reach their academic potential.

It was predicted that the children not receiving any special education services, termed the GenEd group, would earn higher scores on measures of reading, math, and science than children receiving support from a speech-language pathologist, termed the ComDis group. Girls were expected to earn higher scores in each subject than boys. Children from upper- and middle-income households were predicted to earn higher scores in each subject than their lower-income peers.

## METHODS

### Participants

The current investigation employed data from 7946 participants, 3894 males and 4052 females, from the eighth wave of ECLS-K:2011 data collection, taken during the spring of the fourth-grade year^(28)^. The parents of the children participating in the large investigation contained in this dataset provided written consent for participation. The author obtained approval (IRB# 13-053-H) from the IRB of her institution under the exempt category.

Of the 7946 participants, 265 were receiving speech-language therapy, and 7681 were not receiving speech-language therapy. Data were excluded from children who were receiving other special education services to minimize the likelihood that the children in the ComDis group had concomitant disorders. Because these data were obtained via use of an extant dataset, rather than data collected by the author of the current investigation, additional information on speech and oral language ability was not available. Given that schools’ eligibility criteria in the United States are based on federal law^(29)^, it is expected that the children receiving speech-language therapy do present with communication disorders. Additionally, prior research shows that individuals with language disorder tend to be *underidentified*, rather than overidentified^(27)^. The exclusion of children receiving other special education services in the current investigation may have resulted in excluding some children receiving speech-language services; however, the current investigation focused on children with language disorder, and children receiving other special education services may have additional disorders that were not the focus of this study. The inability to confirm the presence of language impairment in the students in the ComDis group and rule out language impairment in the children in the GenEd group is an acknowledged limitation of this investigation; however, federally-mandated eligibility guidelines mitigate the risk of students being erroneously classified into the ComDis group for purposes of this study.

The dataset included an income variable with 18 levels. For the current investigation, the income variable was recoded into three income levels: low, middle, and high. The low-income group included individuals from households earning less than $35,000 annually. The middle-income group’s household income ranged from $35,000-$100,000. Individuals with household incomes above $100,000 comprised the high-income group. The values for these levels were based on the definition of income levels by the Pew Research Center, which was based on data from the United States Census Bureau^(30)^.

### Procedures

The children completed a battery of reading, math, and science assessments individually with a trained examiner, who entered the responses into a computer-assisted interviewing program. On average, children completed this battery in 51 minutes^(31)^. Each subject area was assessed in two stages. A routing section, containing items with a range of difficulty, was administered first, and the child’s score on this section determined whether the child received the low difficulty, middle difficulty, or high difficulty form for each subject. Scores were calculated based on IRT procedures, which enabled the calculation of overall scores for all children, despite children being administered different items. IRT procedures model data using patterns of correct and incorrect responses actually administered and the difficulty, discriminating ability, and “guessability” of the items, based on item-analysis during the piloting phase of the development of the assessment tool. IRT adjusts for the possibility that a child has guessed correct answers and estimates probabilities of children providing correct responses for omitted questions^(31)^. Standardized theta scores were computed for each subject. These scores ranged from -8 to 8^(31)^.

### Measures

The reading assessment for fourth grade built upon the reading assessments created for the earlier rounds of data collection and was based on curricular standards from California, Texas, New Jersey, Florida, and Virginia; the CCSS; and the National Assessment of Educational Progress (NAEP) Reading Frameworks^(32)^. The NAEP Reading Frameworks include poetry, fiction, literary nonfiction, poetry, exposition, argumentation and persuasion, and procedural text. The cognitive targets include *locate and recall*, *integrate and interpret*, and *critique and evaluate*. The Framework uses a systematic approach to vocabulary assessment. The reliability of the reading score in fourth grade is 0.88^(31)^.

The mathematics assessments were based on the Mathematics Framework for the 2005 NAEP^(33)^, the National Council of Teachers of Mathematics *Principles and Standards for School Mathematics*^(34)^, the National Mathematics Advisory Panel^(35)^, the CCSS, and curricular standards from California, Texas, New Jersey, Florida, and Virginia. The Mathematics Framework for the 2005 NAEP focused on five areas: *number properties and operations*, *measurement*, *geometry*, *data analysis and probability*, and *algebra*. The reliability of the mathematics score in fourth grade is 0.92^(30)^.

The science assessments were based on the NAEP Science Frameworks (National Assessment Governing Board^(36)^ and the curricular standards from California, Texas, New Jersey, Florida, and Virginia. The NAEP Science Framework was two-dimensional. The first dimension focused on facts, concepts, principles, laws, and theories in physical science, life science, and earth and space sciences. Four science practices comprised the second dimension: scientific inquiry, life science, physical science, and Earth and space science. The reliability of the science score in fourth grade is 0.83^(31)^.

## RESULTS

Table 1 displays the means for each subject by group, gender, and SES

**Table 1:** Reading, math, and science scores by income, gender, and language impairment status

	**Reading**			**Math**			**Science**		
	**N**	**Mean**	**Standard Deviation**	**N**	**Mean**	**Standard Deviation**	**N**	**Mean**	**Standard Deviation**
ComDis Male Low SES	49	2.43	.77	49	3.09	.77	49	2.24	.78
ComDis Male Mid SES	80	2.85	.47	80	3.62	.68	80	2.72	.66
ComDis Male High SES	48	3.09	.51	48	3.92	.61	48	3.02	.69
ComDis Female Low SES	23	2.54	.47	23	2.88	.72	23	1.74	1.00
ComDis Female Mid SES	38	2.88	.48	38	3.46	.63	38	2.75	.67
ComDis Female High SES	27	3.13	.43	27	3.79	.49	27	2.71	.61
GenEd Male Low SES	1075	2.72	.56	1075	3.34	.66	1075	2.42	.76
GenEd Male Mid SES	1518	3.06	.50	1518	3.74	.58	1518	2.86	.64
GenEd Male High SES	1124	3.23	.47	1124	3.97	.49	1124	3.10	.57
GenEd Female Low SES	1230	2.78	.49	1230	3.19	.66	1230	2.31	.78
GenEd Female Mid SES	1541	3.07	.45	1541	3.52	.60	1541	2.77	.65
GenEd Female High SES	1193	3.25	.45	1193	3.76	.55	1193	2.98	.64

**Caption:** ComDis = children receiving speech-language support services; GenEd = children not receiving speech-language support services; SES = socioeconomic status

A repeated measures ANOVA with a Greenhouse-Giesser correction determined that scores on reading, math, and science differed significantly, with a large effect size, F(1.94, 15359.89)=1306.31, p<.001, η^2^p=.14. Significant interaction effects were found, with small effect sizes, of subject by ComDis status, F(1.94, 15359.89)=3.25, p<.05, η^2^p=.<.001; subject by gender, F(1.94, 15359.89)=27.16, p<.001, η^2^p=.003, and subject by SES, F(1.94, 15359.89)=10.58, p<.001, η^2^p=.003. The four-way interaction of subject by ComDis status by gender by SES was significant, with a small effect size, F(3.87, 15359.89)=3.16, p<.05, η^2^p=.001.

Pairwise comparisons revealed that students performed significantly higher on math than reading and science, and significantly higher on reading than science. Upper-income children performed significantly better than middle- and lower-income children, and middle-income children performed significantly better than lower-income children. Children in the ComDis group earned lower scores than children in the GenEd group. Females earned higher scores than males across income levels and ComDis status for reading. For math and science, the reverse was true, males earned higher scores than females across income levels and ComDis status, with the exception of the middle-income group for mathematics, for which there was no significant gender difference. Females with ComDis and middle SES, males with ComDis with high SES, and females in the GenEd group and low SES had no differences between reading and science scores.

## DISCUSSION

As predicted, and commensurate with prior investigations, fourth graders with ComDis earned lower scores on measures of reading, math, and science than children with typical communication development. Children with high SES outperformed children with middle and low SES, and children with middle SES earned higher scores than children with low SES, as found in prior investigations. The female advantage in reading and language arts found in prior research was supported by the results of this study. However, in the current investigation, males outperformed females in mathematics and science in the lower- and upper-income groups, and no gender difference was found for these subjects for the middle-income group, contrary to the findings of prior studies^(26)^, which did report a female advantage in mathematics and science. The female advantage reported in prior investigations of mathematics and science was less robust than the female advantage for language arts. Given that most participants in most investigations fall into the middle-income category, it is possible that the male advantages in the lower- and upper-income groups were masked by the pattern of differences in the middle-income group when income was not included in the model. Although the current investigation did not find a female advantage in mathematics and science even in the middle-income group, the middle-income group was the one group for which males did not score significantly higher than females in these subjects.

Mathematics was a relative strength for all groups of children. Upper-income females, lower-income children with ComDis, and middle- and upper-income children in the GenEd group earned higher scores in reading than in science. Future investigations aiming to ascertain the mechanisms by which children, including those with ComDis, are earning lower scores in science relative to mathematics and reading scores are needed. As the science education community continues to emphasize the role of language in learning science, it is hoped that improved science achievement in children with and without ComDis will result.

## CONCLUSION

The results of this study highlight the importance of providing support for students with communication disorders across academic subjects. Attention should be paid to supporting children from low-income homes, as these children earned lower scores than those from upper- and middle-income homes for each of the three subjects. Collaborative practice between SLPs and content-area teachers is crucial for ensuring that children with language impairment achieve success in the classroom. Given the role of SLPs in helping all students understand and use the language of the curriculum, SLPs must find ways to partner with other professionals to ensure that low-income children, in particular, are receiving the academic support they need to be successful, even if they have not been diagnosed with language impairment. Additionally, children who have been identified as underperforming in their academic subjects should be referred to SLPs for the screening of language ability to ensure that individuals with language disorder are identified and supported.

It has been noted that the CCSS in the United States have had less success than anticipated in improving teaching and learning, potentially due to limited high-quality instructional materials that help connect the standards with educational practices^(37)^. SLPs have the knowledge and skills to design professional development opportunities for teachers to facilitate broad understanding of the oral language abilities underlying many of the educational standards and techniques to promote growth in the language skills students need to master their academic subjects. The BNCC can be used as a framework for SLPs in Brazil in designing instructional materials and professional development for teachers^(37)^.

Given that mathematics was a relative strength for the fourth graders in this study across ComDis status, income level, and gender, teachers and SLPs could attempt to build on this strength when working on reading and science. For example, using mathematics content when working on reading may benefit students for whom mathematics is a strength. Studies are needed to empirically assess the effectiveness of this approach. Future studies are also needed to identify the types of language support that result in improved outcomes in reading, math and science.
